# Membrane Separation Technology in Direct Air Capture

**DOI:** 10.3390/membranes14020030

**Published:** 2024-01-24

**Authors:** Pavlo Ignatusha, Haiqing Lin, Noe Kapuscinsky, Ludmila Scoles, Weiguo Ma, Bussaraporn Patarachao, Naiying Du

**Affiliations:** 1 Energy, Mining and Environment Research Center, National Research Council of Canada, Ottawa, ON K1A 0R6, Canadanoe.kapuscinsky@nrc-cnrc.gc.ca (N.K.); ludmila.scoles@nrc-cnrc.gc.ca (L.S.);; 2 Department of Biochemistry, Microbiology and Immunology, University of Ottawa, Ottawa, ON K1N 6N5, Canada; 3 Department of Chemical and Biological Engineering, University at Buffalo, The State University of New York, Buffalo, NY 14260, USA; 4 Department of Chemical and Biological Engineering, University of Ottawa, Ottawa, ON K1N 6N5, Canada

**Keywords:** direct air capture, membrane, carbon dioxide, high permeance

## Abstract

Direct air capture (DAC) is an emerging negative CO_2_ emission technology that aims to introduce a feasible method for CO_2_ capture from the atmosphere. Unlike carbon capture from point sources, which deals with flue gas at high CO_2_ concentrations, carbon capture directly from the atmosphere has proved difficult due to the low CO_2_ concentration in ambient air. Current DAC technologies mainly consider sorbent-based systems; however, membrane technology can be considered a promising DAC approach since it provides several advantages, e.g., lower energy and operational costs, less environmental footprint, and more potential for small-scale ubiquitous installations. Several recent advancements in validating the feasibility of highly permeable gas separation membrane fabrication and system design show that membrane-based direct air capture (m-DAC) could be a complementary approach to sorbent-based DAC, e.g., as part of a hybrid system design that incorporates other DAC technologies (e.g., solvent or sorbent-based DAC). In this article, the ongoing research and DAC application attempts via membrane separation have been reviewed. The reported membrane materials that could potentially be used for m-DAC are summarized. In addition, the future direction of m-DAC development is discussed, which could provide perspective and encourage new researchers’ further work in the field of m-DAC.

## 1. Introduction

The terms climate change and climate action have been on the tip of everyone’s tongues for the last few years. Although carbon capture is a promising start, with the carbon budget clock ticking away, it is clear that simply lowering emissions, e.g., capturing CO_2_ from point sources, will not make enough of an impact. It is not long until the carbon budget is depleted, and every additional ton of CO_2_ will need to be managed by the use of negative emission technologies [1,2]. In fact, in order to meet the Paris Climate Agreement goals of preventing a 1.5–2 °C temperature increase over preindustrial levels, 10 GtCO_2_/yr will need to be removed from the atmosphere by the midcentury, increasing to 20 GtCO_2_/yr by the end of the century [3]. Failing to prevent the increase in temperature has a very real social cost. For example, wheat, rice, maize, and soybean represent over 67% of human caloric intake; only a 1 °C increase in temperature will reduce the global production of wheat by 6%, rice by 3%, maize by 7.4%, and soybean by 3.1% [4]. Additionally, changes in weather patterns, acidification of oceans, and melting of polar ice caps place much of the planet’s biodiversity at risk. Therefore, the development of cost-effective negative emission technologies becomes essential to remediate climate change. As one of the negative emission technologies, direct air capture (DAC) describes a process by which CO_2_ is removed directly from the atmosphere rather than from higher concentration point sources. This proves to be quite challenging as the concentration of CO_2_ in the atmosphere is only ~400 ppm [5]. Nonetheless, it is important to develop methods to capture low-concentration atmospheric CO_2_ since capturing all emissions from point sources would fail to accomplish even an 80% emission reduction by 2050 [6], while direct air capture would be able to target the CO_2_ from the billions of small point sources which account for 1/3 to 1/2 of society’s CO_2_ emissions [7].

Currently, sorbent-based DAC technologies are under development at a pilot scale, including solid sorbents and liquid solvents [8,9]. These two technologies rely on absorption/desorption technologies, which require high energy inputs and large location-dependent installations. In addition, these processes often involve the use of chemicals and, therefore, introduce added environmental and safety risks [5,9,10,11,12]. Liquid solvent DAC utilizes contactors where the gas encounters a basic solution. The resulting compounds need intense heating to release captured CO_2_ [8,11,13]. Because of the use of strong bases, the sorbent liquids are usually no more than 30% concentrated, which greatly decreases their binding potential with CO_2_ [14]. The use of strong chemicals, e.g., KOH, also risks a negative environmental impact in the form of spills. In addition, liquid solvents use 1–7 tons of water to capture 1 ton of CO_2_ [9]. Recent research on liquid solvent-based DAC focuses on lowering the consumption of energy and water, e.g., IPDA (3-(aminomethyl)-3,5,5-trimethylcyclohexylamine) liquid to solid carbamic acid conversion for CO_2_ which can capture low (400 ppm) CO_2_ with >99% removal at a lower desorption temperature of 333 K [15]. Solid sorbent methods involve pushing air into a specially designed sorbent until it is saturated. The sorbent is then heated and/or vacuumed to desorb CO_2_ [14]. Solid sorbents have been made of several different materials like metal-organic frameworks (MOFs), mixed metal oxides, poly(ethylenimine) etc. [16]. Metal-organic frameworks are formed through the linkage of organic and inorganic constituents, which form highly structured and microporous materials with high free volumes. The performance of solid sorbent DAC depends on the conditions of the air being processed, including temperature and humidity. High temperatures have been shown to increase energy requirements, leading to a loss of efficiency and an increase in cost [17]. These factors lead to location dependence for DAC installation for both technologies. The source of energy used to power these sorbent-based DAC plants must also be considered when evaluating their level of negative emissions and may limit the location even more [9]. The KOH liquid absorption method reportedly requires 8.81 GJ of natural gas or 5.25 GJ of gas and 366 kWh of electricity for every ton of CO_2_ captured [11]. Solid sorbent energy requirements are around 6 GJ of thermal energy and 1.5 GJ of electricity per ton of CO_2_ [14]. These energy demands arise primarily due to the desorption steps. Although sorbent-based DAC technologies being developed to a plant scale is a great start and is paving a path for DAC, the impact on the environment should not be underestimated, and other environmentally friendly approaches should be explored.

The idea of m-DAC was proposed twenty years ago [18]; however, only recently has a more detailed study been reported that proved that membrane processes could be considered as a new DAC approach [5]. It is a new and rather exciting area of research that shows promise for lower-cost direct air carbon capture and can lower the risk of environmental impact associated with sorption technologies [5]. Theoretically, considering the process only requires energy to blow air through the membranes, advances in membrane materials should drastically decrease the cost of operation, especially given that thermodynamic energy requirements are 20–30 times lower than that of the best DAC methods currently in use [19,20]. However, presently, m-DAC will not be a competitive option to sorbents-based DAC unless major breakthroughs are made in increasing membrane selectivity for the CO_2_:N_2_ gas pair and CO_2_ permeance. Currently, it is widely accepted that m-DAC could play an active role in hybrid system designs that incorporate other DAC technologies. In the past few decades, large amounts of funding have been allocated worldwide for the R&D of membrane-based CO_2_ capture from point sources, and a vast number of published reports related to high permeable polymeric materials for CO_2_ capture membranes could shed light on the selection of DAC membrane materials. Rather than introducing the m-DAC hybrid system, this review gives a perspective of the most recent research on membrane-based direct air capture systems and potential polymeric membrane materials for DAC. Although there is currently very little literature on m-DAC, given its novelty, this review intends to illustrate the need for membrane DAC research and encourage researchers in the field to explore further.

## 2. Feasibility of m-DAC

Although membrane separation is a promising technology for capturing CO_2_ and, to date, a few pilot plants for point source CO_2_ capture have been operated; the pervasive belief is that m-DAC was implausible because the concentration of CO_2_ in the air is only about 400 ppm which leads to an insufficient driving force for CO_2_ permeation through the membranes [21].

Recent achievements in polymer membrane materials with ultrahigh gas permeance and selectivity have exposed the silver lining that membrane separation could potentially be applied in DAC processes. Fujikawa et al. explored the potential of m-DAC based on process simulation with consideration of the state-of-the-art CO_2_ separation membrane performance. The results of the four-stage separation could encourage researchers to explore more realistic membrane performances and process conditions for m-DAC. In their incredibly comprehensive modeling study, they modeled a 4-stage separation process after highly permeable membranes (CO_2_ Permeance of 40,000 GPU (Gas Permeation Unit (GPU) = $3.35\times 10^{-10}\;\frac{\mathrm{mol}}{m^{2}\cdot s\cdot \mathrm{Pa}}$) and CO_2_/N_2_ selectivity of 70) and showed that a CO_2_ concentration of ~30% was achievable at the final stage of a four-stage system with only a membrane area of ~3.2 m^2^/kg CO_2_/day (as a flat sheet) and 16 kWh/kg-CO_2_/day. Their model system assumed a feed pressure of 101.3 kPa and a permeate pressure of 5 kPa and maintained a pre-industrial retentate CO_2_ concentration of ~300 ppm [12].

## 3. Considerations for m-DAC

Direct air capture by membranes depends on several factors, which include membrane properties as well as process parameters.

### 3.1. Permeance

Gas permeance is a measure of the gas transport ability of a material at a given thickness; therefore, it depends on the permeability and thickness of the membrane. Separation by membranes typically follows the Solubility-Diffusion model, which is based on the solubility and diffusion rate of gases going through the membrane [22,23]. Some membranes are capable of facilitated transport mechanisms where carriers react reversibly with CO_2_ selectively, while other gasses only cross by the Solubility-Diffusion mechanism at a lower rate. As a result, these membranes offer high selectivity and gas permeance [24].

(1) $$P=\frac{K}{l}$$

(2) K=S×D

Membranes with a high Solubility-Diffusion relationship and low thickness are candidates for increased permeance based on Equations (1) and (2) where *P* is gas permeance, *K* is permeability (Barrer = $3.35\times 10^{-10}\;\frac{\mathrm{mol}\cdot m}{m^{2}\cdot s\cdot \mathrm{Pa}}$), *l* is membrane thickness, *S* is solubility and *D* is diffusion rate. For power plant post-combustion CO_2_ capture, Merkel et al. concluded that improved gas permeance in membranes is more critical for reducing capture cost than enhanced selectivity [20]. While selectivity is also extremely crucial for DAC, only membranes with high permeance can be considered as an attractive option for DAC. Thinning of membranes is a large area of research for increasing permeance. The thickness of DAC membranes should be reduced to the nanometer level in order to increase membrane permeance significantly because the required membrane area is inversely proportional to permeance. However, it should be noted that studies have shown permeability has a tendency to decrease for many materials in the submicron range, which can lead to a decrease in permeance [25,26].

### 3.2. Selectivity

Membrane gas selectivity (CO_2_/N_2_) is of great interest for m-DAC design, given that nitrogen is the largest component of the earth’s atmosphere and the CO_2_ concentration is only about 400 ppm. Ideal gas selectivity is defined as the ratio of CO_2_ to N_2_ permeability, as shown below.

(3) αCO2N2=KCO2KN2

where *α* is the selectivity, $K_{{CO}_{2}}$ is CO_2_ permeability and $K_{N_{2}}$ is N_2_ permeability in single gas tests [27]. The determination of selectivity becomes more complicated when it comes to mixed gas testing. Low selectivity leads to a higher migration of unwanted gases and a lower concentration of CO_2_ in the permeate. As a rule of thumb, permeance should be maximized to decrease membrane area [12], but selectivity should be optimized as membranes with a selectivity that is too high require more membrane area and show little benefit in CO_2_ purity. Merkel et al. compared two membranes for point source CO_2_ capture, the “best case” membrane and membrane B. The “best case” membrane had a CO_2_ permeance of 1000 GPU and a CO_2_/N_2_ selectivity of 50, while membrane B had a CO_2_ permeance of 1000 GPU and a CO_2_/N_2_ selectivity of 200. They found that the best-case membrane produced a permeate with 46% CO_2_ at 2.1 MM m^2^, while membrane B yielded 55% CO_2_ with an area nearly three times larger at 5.7 MM m^2^ [20]. The concentration of CO_2_ in the permeate increases greatly with selectivity until a selectivity of about 30 when it begins to plateau. The selectivity also decreases the energy required for vacuuming, which similarly plateaus. Fujikawa et al. showed that a membrane with a CO_2_ permeance of 10,000 GPU and a CO_2_/N_2_ selectivity of 50 could reach 66.7% CO_2_ with only 4.63 m^2^/kg CO_2_/day and ~12.3–16 kWh/kg-CO_2_/day at a pressure ratio of 50 using a feed pressure of 110 kPa and a permeate pressure of 2 kPa. The membrane area required increases with selectivity almost linearly while the benefits in CO_2_ concentration, energy requirement, and CO_2_ emission related to the energy production decrease with each 10-step selectivity increase [12].

Although less important than the previous two parameters, CO_2_/O_2_ selectivity should also be considered depending on downstream applications of captured CO_2_ as processes like the reduction of CO_2_ to CO and CH_4_ are typically hindered due to the oxidative pressures of O_2_ [12].

### 3.3. Pressure Ratio

The pressure ratio is feed pressure divided by permeate pressure.

(4) $$\varphi =\frac{p_{f}}{p_{p}}$$

(5) $$X_{p}\leq X_{f}\times \varphi$$

where *p_f_* and *p_p_* are feed and permeate pressures respectively. Equation (5), where *X_p_* is the mol fraction in the permeate and *X_f_* is the mol fraction in the feed, shows that the mole fraction of CO_2_ on the permeate side is limited by the pressure ratio independently of other parameters, notably selectivity [12,23]. For flux across the membrane in the desired direction, the partial permeate CO_2_ pressure cannot exceed the partial feed CO_2_ pressure in order to maintain a favorable cross-membrane pressure gradient. Because the mole fraction in the permeate is limited proportionally by the pressure ratio, a higher pressure ratio leads to greater permeate CO_2_ concentrations. The largest effect of pressure ratio on CO_2_ permeate concentration is observed under φ=30, after which the effect begins to plateau [12]. Vacuuming processes have been shown to be more energy efficient than feed compression systems for gas separation [23].

Fujikawa et al. studied the effect of pressure ratio on m-DAC via process simulation with the same CO_2_ retentate concentration (~300 ppm) and the same membrane (permeance of 40,000 GPU and selectivity of 70) (Table 1); however, the pressure ratio is different. When the pressure at the permeate side is 4 kPa (*φ* = 25), the final CO_2_ concentration can exceed 40%. Membrane area and CO_2_ emissions related to the energy production decrease from 3.19 to 2.6 m^2^/kg-CO_2_/day and 0.6 to 0.54 kgCO_2_^emitted^/kgCO_2_^captured^, respectively, when the pressure ratio is raised from 20 to 25. It is important to note that at each stage, the energy and membrane area required falls dramatically when compared to the first stage. This illustrates the importance of process parameters in addition to material properties like permeance and selectivity.

### 3.4. Stage Cut

Stage cut is defined as permeate flow divided by feed flow.

(6) $$\emptyset =\frac{f_{p}}{f_{f}}$$

where ∅ is Stage cut and *f_p_* and *f_f_* are permeate and feed flows, respectively [12,28]. A high feed flow leads to a lower change in feed gas concentrations, which makes the difference in driving forces for unwanted gases more negligible when compared to CO_2_. As a result, the purity of the permeate is higher, but the recovery % is lower as less CO_2_ crosses at a low-stage cut. Decreasing the flow rate of the feed increases the amount of CO_2_ that crosses but, in doing so, increases the driving force for other gases. Therefore, a higher stage cut leads to a lower purity but a higher recovery % of CO_2_. Membranes with higher areas lead to greater stage cuts, lower purity, and a higher % CO_2_ recovery [12]. All of these parameters should be fined-tuned for m-DAC systems depending on the final goal, whether that be total recovery or a high purity for downstream applications.

In addition, these parameters may vary at each stage of a multi-step separation system. Energy requirements for installation, operation as well as manufacture of membranes must also be considered. Due to the infancy of m-DAC technology, there are very few parametric and gas module separation studies available. With that being said, several models that take these parameters into account have shown the scope of the possibility of m-DAC. High selectivity membrane material (HPM)with a CO_2_ permeance of 2500 GPU and CO_2_/N_2_ selectivity of 680 has been shown to be capable of a capture purity of nearly 20% in a single stage at a pressure ratio around 0.02, and commercial Polaris membranes (CO_2_ permeance of 2000 GPU, CO_2_/N_2_ selectivity of 30) can reach 50% with two stages [29]. HPM showed an exponential increase in CO_2_ capture purity when decreasing the pressure ratio from 0.02, while no significant effect was observed for the Polaris membrane due to a negligible effect from concentration polarization [29]. For m-DAC, HPM and Polaris membranes required 3000 and 18,000 kWh/ton at one stage, respectively, to achieve 20 and 2.5% purity [29]. The study outlined the importance of a low-stage cut for improving the recovery ratio [29]. Although m-DAC CO_2_ concentrations are not commonly tested, Lee et al. showed their ionic liquid + graphene oxide PIL-IL-GO facilitated transport membrane could produce a purity of 32% at 410 ppm CO_2_ using only a single stage at 1 bar (1 bar = 100 kPa) of feed pressure [30]. Under these conditions, the membrane displayed a permeance of 3090 GPU and a CO_2_/N_2_ selectivity of 1180 [30]. Unfortunately, the stage cut and pressure ratio were not reported. Proposed multistage membrane separation models have shown improved CO_2_ separation with each stage and notably outline that the first stage has the highest energy, area, and emission values, after which each sequential stage shows an exponential decay [12].

Although very few papers have reported membrane separation under m-DAC conditions, the former examples indicate real promise and should encourage more widespread testing of membranes with atmospheric CO_2_ capture in mind.

## 4. Potential Membrane Materials

**Figure 1:**
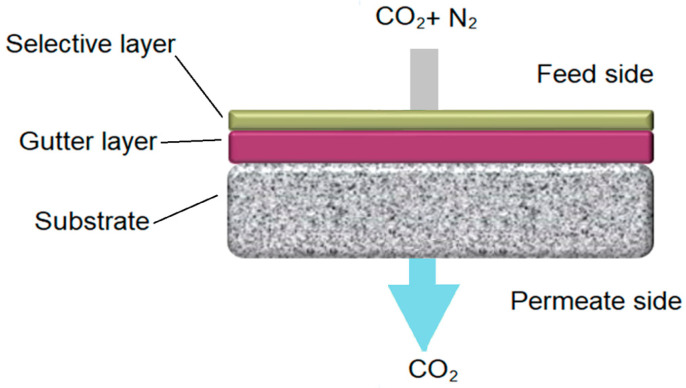
Sample diagram of typical gas separation membrane layers.

According to the concern above, the thickness of polymeric selective layers for DAC needs to be less than 1 µm. Hence, a typical membrane for DAC should consist of a thin selective layer deposited on an intermediate gutter layer on a porous substrate support, as shown in Figure 1. The gutter layer serves to protect from the penetration of the selective layer material into the porous support [25]. Highly permeable polydimethylsiloxane (PDMS) is frequently used as the gutter layer, and polyacrylonitrile (PAN) is used as the substrate [25,31,32,33]. Although each layer of material introduces transport resistance, the PDMS and PAN are so porous that their effect is negligible. Selective layers are manufactured by several different methods, including spin coating, dip coating, roll coating, blading, solution casting, dry-jet wet spinning (hollow fiber), and spray coating, with spin coating being the most common method for ultrathin membranes [32,34,35,36,37].

The selective layer is the most vital element of composite membranes and is modified with permeance and selectivity in mind. Fujikawa et al. recommend membranes with a CO_2_ permeance of >10,000 GPU and CO_2_/N_2_ selectivity of >30 for m-DAC. However, most current membranes fail to reach these specifications [12]. In the last decade, numerous promising polymeric membranes have stood out. Materials such as polymers of intrinsic microporosity (PIMs), polymers with ethylene oxide/ethylene glycol groups, mixed matrix membranes (MMMs), and facilitated transport membranes might meet the standards set by Fujikawa if their thickness falls below 1 µm as the permeance could be increased. Inorganic membranes have been explored in the past but often suffer drawbacks due to price and scalability [26].

In the last two decades, researchers have tried different approaches to increase permeability and selectivity, as well as to reduce the aging of membrane materials, for instance, introducing more rigid units into the polymer structure, post-modifying polymer structure with different functional groups, and adding functionalized nanofillers to membranes creating MMMs [38]. In this paper, we summarize the potential polymeric membrane materials for DAC. These materials with high permeability/permeance and/or high CO_2_/N_2_ selectivity near or above the 2008 Robeson upper bound are mainly applied to CO_2_ capture from point sources (Table 2). However, they could still be good starting candidates for further m-DAC development [39].

### 4.1. Copolymers

Various copolymers with high CO_2_ permeability and CO_2_/N_2_ selectivity have been explored. Their structural designs focus on adding rigid structures to reduce interchain packing and create a microporous structure, in addition to frequently introducing ethylene oxide and ethylene glycol groups to enhance CO_2_ affinity.

Aging is the major drawback of these microporous polymers [38,71]. Aging refers to the thermodynamic stabilization of the membrane polymer packing, which leads to a collapse of pores and a loss of free volume, resulting in a decrease in permeability. This phenomenon is observed after short periods of time and at accelerated rates with thinner membranes [25,34,72,73]. Membranes also suffer from plasticization, where CO_2_ sorption at high pressures increases polymer chain movement. This leads to an increased permeability for all gasses due to an increase in free volume and, consequently, a decrease in selectivity [74].

Introducing rigid and bulky units into the polymer chain can efficiently increase CO_2_ permeabilities with or without aging while still maintaining moderate selectivity due to their special chain rigidity. PIMs are a good example of polymers that successfully increase permeability by introducing rigid structures into the polymer chain. PIMs are usually composed of two components: (i) a structural unit that possesses concavities and introduces a site of contortion into the polymer chain and (ii) a linking group (e.g., derived from dibenzodioxin or imide formation) that fuses the structural units together during polymerization and leads to inefficient packing and high free volume [25,73,75]. Some of these polymers offer high permeability as well as cheap facile manufacture. The main building blocks of PIMs are (i) spirobisindane (SBI); (ii) phenazine; (iii) ethanoanthracene (EA); (iv) triptycene (Tridp); (v) benzotriptycene (BTrip); (vi) spirobifluorene (SBF); (vii) Tröger’s base (TB); and (viii) tetraphenylethylene etc. [76]. Polyimides of intrinsic microporosity (PIM-PIs) can also be prepared when structural PIM motifs containing rigid and bulky contortion sites are introduced into polyimide backbones. The structural design can be manipulated in many different ways depending on the location of the contortion site, for example, either in dianhydride or diamine monomers for PIMs-PIs. The method in which PIMs are synthesized has been shown to have an effect on chain packing, membrane structure, performance, and aging.

**Figure 2:**
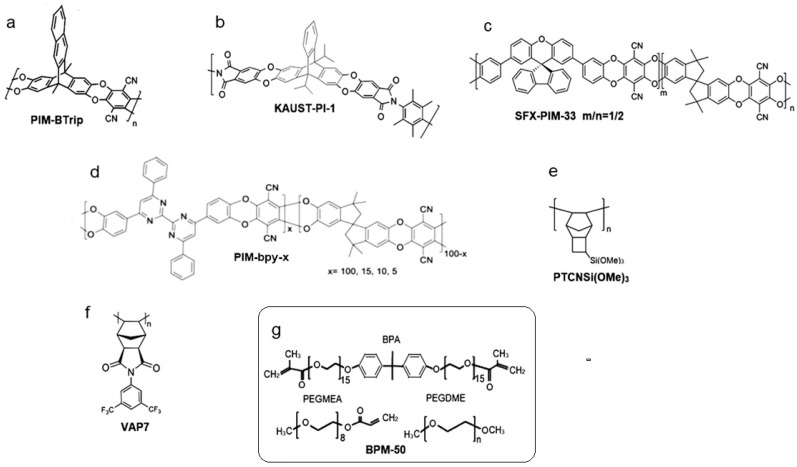
Chemical structures of high permeable copolymers, with CO_2_ permeabilities ≥ 1000 Barrer or with CO_2_/N_2_ Selectivity’s ≥ 30 at temperatures ≤ 35 °C. (**a**) PIM-BTrip [41], (**b**) KAUST-PI-1 [40], (**c**) SFX-PIM-33 (m/n = 1/2) [42], (**d**) PIM-bpy-x [77], (**e**) PTCNSi(OMe)_3_ [45], (**f**) VAP7 [44], (**g**) BPM-50 [43].

PIMs containing Trip and BTrip (Figure 2a) units exhibit excellent CO_2_ permeabilities (up to ~22,000 Barrer) [41]. Their highly rigid structure leads to observed ultra-microporosity, which facilitates the transport of smaller gas molecules whilst increasing the activation energy for larger molecules like N_2_. Experimentally, PIM-BTrip has been shown to have a high diffusivity, leading to greater permeability whilst maintaining selectivity due to the selective molecular sieving based on kinetic diameter [41]. KAUST-PI-1 is another example of a co-polymer that boasts high permeability due to its rigid backbone structure, which effectively leads to the formation of ultra-porous structures (Figure 2b). It was also observed that the choice of bridgehead group had a significant effect on the permeability of KAUST-PI-1. The bulky isopropyl bridgeheads and methyl-substituted diamines increased intrachain rigidity and improved CO_2_ permeability 4-fold [40]. Co-polymer designs with bulky groups in mind, like SFX-PIMs (Figure 2c), have shown CO_2_ permeabilities comparable to the PIM-1 standard but with improved CO_2_/N_2_ selectivity around 30 [42]. PIM-bpy-x gas separation performance was enhanced by incorporating bulky structures into the polymer through the polycondensation of a tetraphenyl bipyrimidine monomer (Figure 2d). PIM-bpy-x exhibited excellent gas separation performance (4234 barrer)—a 21% improvement in CO_2_ permeability. The increase in CO_2_ permeability is due to the affinity of the N-rich bipyrimidine units for CO_2_ [77].

High permeable polymers synthesized from different modified monomers have also shown the improvement of gas separation properties. Grazia Bezzu et al. conducted a combined simulation and experimental study to investigate the effect on polymer microporosity and gas permeability of PIM-SBFs by placing simple substituents such as methyl, t-butyl, and fused benzo groups onto spirobifluorene monomers. It was shown that methyl or t-butyl substituents both cause a large increase in gas permeabilities, with four methyl groups enhancing the concentration of ultramicropores (<0.7 nm), which contribute to selective gas transport. The t-butyl substituents lower selectivity by generating a greater concentration of larger, less selective micropores (>1.0 nm) due to their size [78]. Alentiev et al. wonderfully demonstrated the variability that can occur with subtle modifications of side groups on the monomers (Figure 2e). Following this strategy of monomer substituent modification, new high molecular weight metathesis and addition polynorbornenes with (AlkO)_3_Si-groups of different lengths (Alk = Me, Et, n-Pr, n-Bu) were synthesized. These polymers, with similar scaffolding, consist of two parts—glassy (rigid polymer main chains) and rubbery (flexible side chains), which allowed dramatic tuning of polymer properties by the modification of polymer main chain structures and the length of trialkoxysilyl side groups. PTCNSi(OMe)_3_, for example, showed a selectivity of 35.7 while increasing the length of substituents with PTCNSI(OEt)_3_ led to a selectivity drop down to 21.3 [45]. The drop in selectivity was likely due to the longer chains creating larger pores. Nazarov et al. performed a similar study exploring several modified monomers that formed vinyl-addition polymers. VAP7 was the most promising for DAC application (Figure 2f). VAP7′s performance can be attributed to the trifluoromethyl groups, which hinder inter-chain interactions, leading to an increase in free volume due to a decrease in favorable chain packing [44].

Ethylene oxide and ethylene glycol groups have been exploited in membrane design due to their high affinity for CO_2_ by Lewis base interactions, which often lead to a higher selectivity for CO_2_ [79,80]. Membranes are not made solely of polyethylene glycol due to the high crystallinity induced by strong hydrogen bonding, which is not attractive for gas permeability. Both Polaris and Polyactive (shown in Table 2) are commercial copolymers containing PEO groups [80,81]. Both of these PEO-containing copolymers show CO_2_/N_2_ selectivity over 30 and respectable CO_2_ permeance [46,47]. Pebax 1657 is also a promising PEO-containing commercial polymer that is frequently used in MMM applications. BPM-50 (Figure 2g), an oxygen-containing copolymer that was formed through the UV polymerization of PEGMEA, BPA, and PEGDME, boasts an impressive CO_2_ permeability of 4883 Barrer and CO_2_/N_2_ selectivity of 43. In addition, the method of preparation for BPM-50 is clean, rapid, and solvent-free [43].

**Figure 3:**
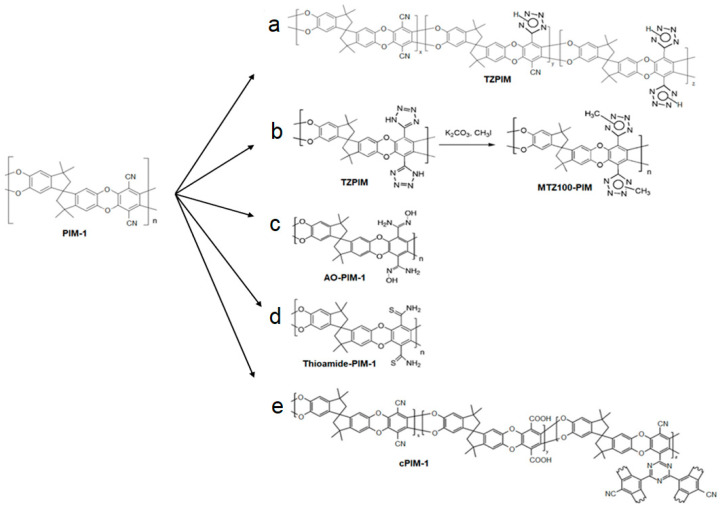
Chemical structures of copolymers with post modification (CO_2_ permeabilities ≥ 1000 Barrer or with CO_2_/N_2_ selectivity’s ≥ 30 at temperatures ≤ 35 °C). (**a**) TZPIM [48], (**b**) MTZ100-PIM [50], (**c**) AO-PIM-1 [49], (**d**) Thioamide-PIM-1 [51], (**e**) cPIM-1 [71].

Another effective approach to significantly improving the permeability and selectivity is post-modifying copolymers. The functionalization of promising copolymers allows for further enhancements to gas separation performance (Table 2). Taking TZPIM as an example (Figure 3a), the tetrazole structures from the modification of nitrile groups in PIM-1 raise the affinity for CO_2_, hence increasing the single gas selectivity of CO_2_/N_2_ to 30. These tetrazole groups also serve to increase rigidity through hydrogen bonding, which in turn increases plasticization resistance from CO_2_ [48]. Further modifying TZPIM to MTZ100-PIM (Figure 3b) increases solvent processability over TZPIM-1, which is of critical importance for large-scale membrane fabrication [50]. MTZ100-PIM showed a significant change in performance between pure and mixed gas permeation testing. Under pure gas testing, MTZ100-PIM showed a CO_2_ permeability of 1391 barrer and a CO_2_/N_2_ selectivity of 22.2. Conversely, mixed gas testing with a 2:8 CO_2_:N_2_ per volume mixture yielded more impressive results with a CO_2_ permeability 2057 barrer and a CO_2_/N_2_ selectivity of 41.6. This behavior is attributed to a suppression of N_2_ permeability as condensable CO_2_ occupies sorption sites far more than the non-condensable N_2_, reducing the rate of passage for N_2_. AO-PIM-1 is an excellent example of the common observations that come with functionalization (Figure 3c). The oxygen and nitrogen groups lead to more interchain interactions, which causes a net decrease in microporosity and the formation of more narrow pores. This, in turn, results in a decrease in CO_2_ permeability and an increase in CO_2_/N_2_ selectivity to 35 due to the electronegative groups interacting favorably with CO_2_ [49]. Thioamide-PIM (Figure 3d) continues this trend with lower CO_2_ permeability but higher CO_2_/N_2_ selectivity (30.3) when compared to PIM-1 [51]. Yu et al. recently tested di-substituted PIM-1 (D-cPIM-1) and branched PIM-1 (B-cPIM-1) containing carboxylic acid groups (Figure 3e). They found that the 70% hydrolyzed di-substituted PIM-1 showed a CO_2_ permeance of 7700 GPU and CO_2_/N_2_ selectivity of 56. More importantly, although the branched hydrolyzed PIM showed a lower CO_2_ permeance of 3200 GPU, it showed no decrease in permeance after 60 days, indicating the branched structure may be vital for aging resistance [71]. These hydrolyzed PIMs have also been investigated for increasing CO_2_ permeability due to quadrupole interactions, as further illustrated by cPIM-1 [52].

PIM and several polymers with similar structures are often treated with ethanol or methanol to remove residual solvent from casting and increase the diffusion coefficient, thus increasing permeability [82,83]. UV treatment has also been shown to increase performance due to chain scission and oxidation of the polymers [84].

Although the CO_2_/N_2_ gas separation performance of some polymers synthesized via thermal or UV treatment meets our summary criteria showing increased separation properties, e.g., carbon molecular sieves, TOX-PIM-1 [85], PIM-300 [86], and PIM-1-UV/Ozone [84], the high fabrication temperature, brittleness of thin sheets and poor solubility after treatment could limit their feasibility in m-DAC.

### 4.2. MMMs

The introduction of filler materials into polymeric membranes integrates their advantageous properties and provides a feasible approach to fabricating membranes for DAC [87,88,89]. These fillers offer a wide range of functionality due to their varying structures and often have low cost. Most added fillers help with molecular sieving due to their innate structure or, based on their dispersion/interaction with the polymeric material, can help create free volume and improve the gas-membrane affinity to provide effective solutions for overcoming the performance trade-off effect [57]. These structures can range from 0D to 3D with varying complexities and molecular interactions with gas molecules [90].

**Figure 4:**
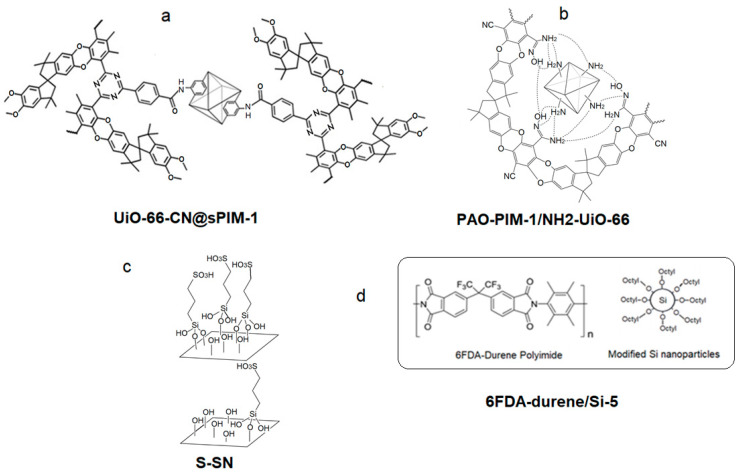
Examples of high permeable MMMs with MOF and Silica nanoparticles (CO_2_ permeabilities ≥ 1000 Barrer or with CO_2_/N_2_ Selectivity’s ≥ 30 at temperatures ≤ 35 °C). (**a**) UiO-66-CN@sPIM-1 [57], (**b**) PAO-PIM-1/NH_2_-UiO-66 [61], (**c**) S-SN [95], (**d**) 6FDA-durene/Si-5 [53].

Amongst the crowd of MMM fillers, MOFs stand out as they consistently have positive effects on membrane performance depending on their loading [57,91,92,93]. For example, UiO-66-CN covalently linked with s-PIM boasted a CO_2_ permeability of 16,121 Barrer and CO_2_/N_2_ single gas selectivity of 27.0. In addition, when tested under 1:1 CO_2_:N_2_ by volume, the selectivity increased to 53.5 (Figure 4a). This is explained by the high porosity and rich sorption sites in the 3-D MOF domain, where CO_2_ is competitively adsorbed over N_2_ [57]. UiO66-NH_2_ dopped with Ag^+^ leads to great increases in gas separation characteristics when tested under 1:9 CO_2_:N_2_ by volume as Ag^+^ raises the hydrophilicity and facilitates CO_2_ transport while also leading to an increase in free volume, which characteristically increases permeability to >15,000 Barrer with selectivity 30 [94]. Wang et al. reported a hybrid membrane containing UiO66-NH_2_ in PAO-PIM (Figure 4b). The amidoxime and amine groups tend to form hydrogen bonds, creating a hydrogen bond network between the two phases, which raised the CO_2_ permeability from 2902 to 3825 Barrer due to an increase in the diffusivity coefficient while still maintaining a respectable selectivity [61].

3D MOF fillers are not the only inorganic fillers being explored for mixed matrix membranes. The addition of silica nanoparticles is an efficient strategy for boosting the initial gas permeability and suppressing physical aging. By incorporating 0.05 wt% sulfonic acid-functionalized silica nanosheets (S-SN) into the PIM-1 polymer, the resulting membrane showed a 40% higher CO_2_ permeability and 22% enhancement in CO_2_/N_2_ selectivity. 150-day-aged freestanding PIM/SN1 and 28-days-aged TFN PIM/S-SN0.05 showed 70% and 5 times greater CO_2_ permeability than the pure thick PIM-1 and TFC PIM-1 membranes, respectively [35] (Figure 4c). Similarly, Nafisi et al. studied the effect of silica nanoparticle loading in both Pebax and 6FDA-durene matrices. They found that the integration of silica nanoparticles raised the permeability in both cases, more dramatically with 6FDA durene (Figure 4d). They observed an increase in Fractional Free Volume (FFV) with filler loading; FFV increases as the density of the membrane decreases. The more drastic increase in permeability observed with 6FDA durene is hypothesized to be due to its glassy nature; therefore, the presence of nanoparticles has a greater effect on the FFV [53].

Other inorganic fillers, such as synthetic zeolite—SAPO-34, can also largely increase the CO_2_ permeability of MMMs [95]. Similarly, SAPO-34 raises the free volume of polymer membranes, leading to an increase in permeability and raising selectivity when selectively sieving molecules based on kinetic diameter [58]. It was reported that a PDMS-SAPO-34 (PM-30 wt%) membrane exhibited a CO_2_ permeability of 5753 Barrer with an ideal CO_2_/N_2_ selectivity of about 31 at 2000 kPa (20 bar) and 25 °C, which surpassed the 2008 Robeson upper bound for CO_2_/N_2_ separation.

**Figure 5:**
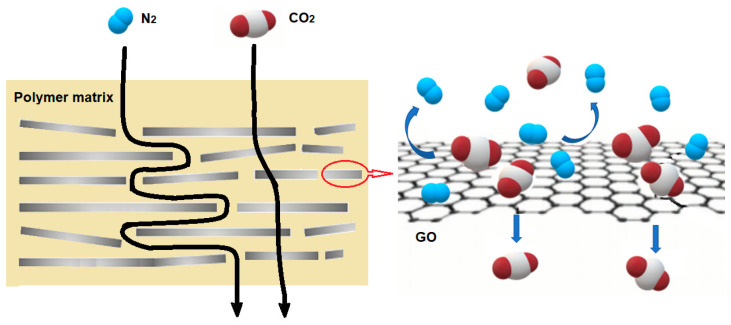
Schematic of the mechanism for gas travel through MMMs with GO fillers.

Also, a large number of novel inorganic fillers could not only help tune the free volume of the polymer matrix but also create the coefficient path. Carbon nanotubes and graphene oxide (GO) nanosheets are examples of such fillers. Efficient CO_2_ transport pathways were constructed by the homogeneous dispersion of carbon nanotubes (CNTs) and GO within MMMs, leading to both enhanced permeability and selectivity. Li et al. showed that mixing both carbon nanotubes and GO fillers into Matrimid membranes displayed a synergistic relationship [96]. The extraordinarily smooth walls of CNTs acted as a highway for high permeability, whereas the graphene oxide nanosheets acted as a selective barrier to create a more tortuous diffusion pathway for N_2_, meanwhile providing a more selective path for CO_2_ through interactions with the hydroxyl and carboxyl groups on the GO surface (Figure 5). The membrane combining 5 wt% of CNTs and 5 wt% of GO (Matrimid^®^-CNTs/GO-5/5) displayed optimum performance with a CO_2_ permeability of 38.07 Barrer, a CO_2_/N_2_ selectivity of 81.00. Compared to pure Matrimid^®^ membranes, the CO_2_ permeability and CO_2_/N_2_ selectivity of the Matrimid^®^-CNTs/GO-5/5 membranes were increased by 331% and 147%, respectively. Although the reported 38.07 Barrer is still much lower than the expected permeability of CO_2_ for m-DAC, the method inspires future research on designing high-performance membranes for DAC. More effort needs to be put into choosing the proper polymer matrix. Furthermore, to achieve high performance, ideal polymer–filler interface morphology and homogenous filler dispersion have to be realized. These challenges can be addressed by appropriate screening or modification of fillers to balance the polymer–filler, and filler–filler interactions [97].

Besides inorganic fillers, organic hollow nanoparticles have great promise for creating new highly permeable membranes. Ding et al. fabricated hollow polyamide nanoparticles (HPN) with a dense shell via interfacial polymerization in a surfactant-free microemulsion. The hollow nanoparticles combined with liquid acrylate monomers to form mixed matrix membranes via UV-induced photo-polymerization. The nanoparticles with an average diameter of 42 nm dispersed uniformly in the membranes. The CO_2_ permeability and CO_2_/N_2_ permselectivity of the MMMs both increased as the nanofiller loading increased. The gas permeation and separation performance exceeded the Robeson upper bound line with a maximum CO_2_ permeability of 1898 Barrer and a maximum CO_2_/N_2_ permselectivity of 43.9 at 1 wt% nanofiller loading. The improvement mainly arose from the increase in the CO_2_ solubility, the N_2_ diffusivity, and the CO_2_/N_2_ solubility selectivity [62].

**Figure 6:**
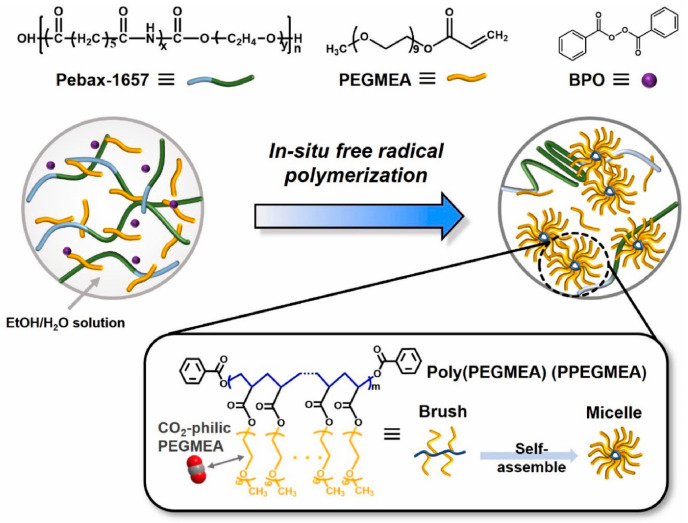
Schematic of Pebax/PPEGMEA membranes obtained via the free radical polymerization of PEGMEA in Pebax in the presence of BPO. Reprinted with permission from Ref. [99]. Copyright 2022 Elsevier.

In addition, several novel molecular-scale fabrication methods render highly efficient polymeric composites and could be potentially applied for m-DAC. Instead of using traditional substrates and gutter layers, Ashtiani et al. developed membranes with carbon nanotube support structures and a ZIF-8 gutter layer (zeolitic imidazolate frameworks-ZIF). The surface was spray-coated with PDMS, which helped fill any gaps or interfacial defects. Due to the ZIF-8′s metal sites, CO_2_ was effectively permeated through feeble electrostatic fields and the quadrupole moments of CO_2_ [60]. The in-situ micelle-induced blending approach is another significant step toward realizing high-performance CO_2_ separation membranes. Seong et al. reported highly CO_2_-permeable membranes obtained by blending in situ self-assembled micellar-structured poly(poly(ethylene glycol) methyl ether acrylate) (PPEGMEA) with poly(ether-block-amide) (Pebax) (Figure 6). The Pebax/PPEGMEA (30/70 *w*/*w*) blend membrane comprised highly CO_2_-philic PPEGMEA micelles, which appreciably increased the d-spacing of the Pebax matrix. Consequently, the blend membrane containing a high molecular weight of PPEGMEA exhibited an unprecedented CO_2_ permeability enhancement of 1054% compared to the pristine Pebax membrane while maintaining good CO_2_ selectivity relative to N_2_ because of the enriched polyethylene glycol moieties. This excellent separation performance was maintained for 100 h up to 10 atm, validating the good long-term separation performance of the membrane [98]. Furthermore, the membrane exhibited good mechanical strength and plasticization tolerance. Similarly, membranes formed through the radical polymerization of PEGMEA, PEGDA, and PEGDME presented CO_2_ permeability of 2980 Barrer and CO_2_/N_2_ selectivity of 45.7 with stability over 500 h [99]. Gel mixed matrix composites provide a class of membranes with high CO_2_ permeability and stability performance. The fillers incorporate alongside small molecule liquid substances to increase free volume [100]. Chen et al. prepared EM400/MIL-101(Cr)–NH_2_ MMMs; the addition of MIL-101(Cr)–NH_2_ improved the mechanical properties of the membrane and increased the selectivity of CO_2_/N_2_. The EM400/MIL-101(Cr)–NH_2_ MMMs were further modified with tripropionin. The tripropionin greatly improved the CO_2_/N_2_ separation performance of the g-MMMs by increasing the FFV and the CO_2_ solubility coefficients at the same time. The CO_2_ permeability was increased from 213 to 1182 Barrer when compared to the EM400/MIL-101(Cr)-NH_2_ MMMs without TPP. This kind of gel-MMM also showed stability over 168 h of aging testing [100]. An alternative study tested SPEEK/MIL-101 (Cr) and SPEEK/S-MIL-101 (Cr) membranes at 40% loading under humidified conditions. Under these conditions, both membranes showed permeability improvements from 30 to 1623 Barrer and 35 to 2064 Barrer, respectively. Although the selectivity for SPEEK/MIL-101 (Cr) membranes did not change from 40, the selectivity did improve from 41 to 53 in the case of SPEEK/S-MIL-101 (Cr). The proportionality between permeability and water content was attributed to an increase in pore swelling, while the increase in selectivity can be explained by the decreased level of transport resistance for CO_2_ with water as compared to N_2_ [56].

### 4.3. Facilitated Transport Membranes

**Figure 7:**
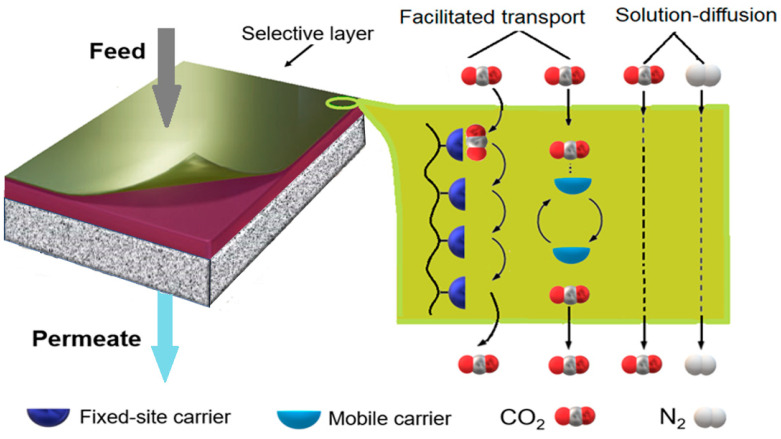
CO_2_/N_2_ separation through the facilitated transport membrane [12,104].

Finally, facilitated transport membranes are unique in their exceptionally high selectivity for CO_2_ compared to typical solubility/diffusion membranes due to reversible reactions between the carriers in the membrane and CO_2_. The carriers can be divided into fixed or mobile carriers (Figure 7). These reversible reactions require water to occur, setting a dependence on the humidity of the feed gas [101]. When considering m-DAC, the need for humidity can lead to tighter requirements for installation limited to humid areas or introduce higher operating costs and water requirements for humidifying the feed gas. With that being said, PIL-IL-GO membranes have been tested under m-DAC conditions of 410 ppm CO_2_ and showed CO_2_ permeances between 3000 and 4000 GPU with very high CO_2_/N_2_ selectivity (>1000) [30]. There are various carrier structures, but most are based on amines. For example, P(DADMACA-co-VAm) membranes containing primary amino groups, carbonate groups, and quaternary ammonium groups show synergistic increases in membrane separation performance over their homogeneous counterparts (PVAm/PDADMACA) with a CO_2_ permeance of 1842 GPU and a CO_2_/N_2_ selectivity of 160 [63]. In addition, PVA membranes have been modified with both SiO_2_ nanofillers and [bmim][Tf_2_N] ionic liquid to produce membranes with a CO_2_ permeance of 3016 GPU and a CO_2_/N_2_ selectivity of 62 [102]. The use of ionic liquids may not be the best practice as they can be high-cost and harmful to the environment and living organisms, therefore eliminating one of the advantages of m-DAC over the other sorption technologies [103].

## 5. M-DAC Application

**Figure 8:**
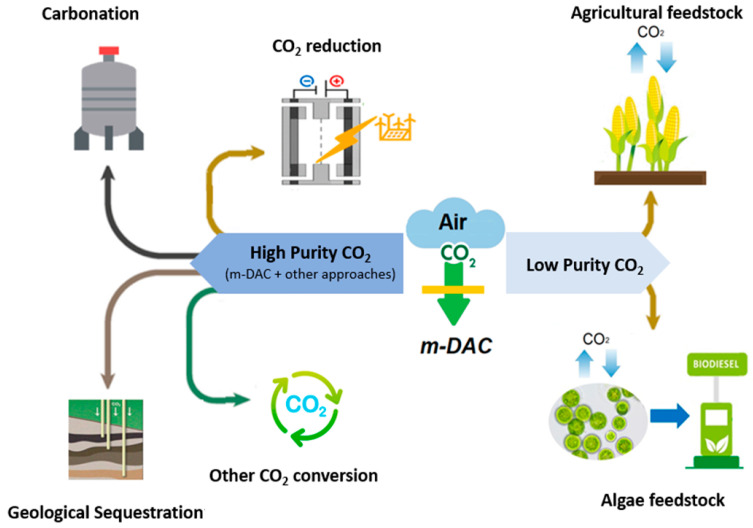
Examples of post-capture utilization pathways for CO_2_ depending on purity.

Once CO_2_ is captured by DAC, it can be stored or recycled for use in several different downstream applications, as summarized in Figure 8. One important parameter for any CO_2_ utilization after capture is the percent purity of CO_2_. The most common method of CO_2_ storage is geological sequestration; generally, geological storage of CO_2_ requires CO_2_ gas at a high purity (more than 98%) [5,105]. Currently, a purity over 90% is not possible to achieve by m-DAC efficiently in 1 stage; therefore, geological storage is an unlikely application of m-DAC without several stages or pairing with other CO_2_ capture approaches [5,12,16]. A simulation-based study using a membrane with a CO_2_ permeance of 1850 GPU and CO_2_/N_2_ selectivity of 80 for hybrid membrane cryogenic (HMC) capture showed a 9% reduction in capture cost at a capture ratio of 85% compared to conventional monoethanolamide (MEA) carbon capture [106,107]. Similarly, another HMC process using a membrane with a CO_2_ permeance of 1000 GPU and CO_2_/N_2_ selectivity of 100 was shown to reduce the energy requirement compared to MEA capture from 4.409 GJ_th_/t_CO2_ to 3.25 GJ_th_/t_CO2_ from a stream of 15% CO_2_ and a pressure ratio of 11.11 achieving a capture ratio of 85% and a purity over 89% [107,108]. Unfortunately, there is a lack of research on hybrid separation systems using membranes alongside other DAC methods, which have the possibility for efficient cost reductions. A recent engineering parametric study, which investigated the impact of material performance, process design, and operating conditions on membrane-based DAC, shed light on the future of this technique. The study concluded that with existing commercial membrane materials, the maximal CO_2_ output concentration from a single-stage separation would be ~2%. A similar two-stage process could increase this to ~50%, noting that O_2_ and water would be co-permeated. High-performance materials (not commercialized) could achieve ~12% in a single stage and up to ~99% in two stages [16]. For these high-performance materials in a two-stage process, costs and energy fall in the affordable range, e.g., 103 to 104$ per tCO_2_ with a first-order optimized energy requirement of 101 GJ per tCO_2_ [16]. Currently, multi-stage membrane modules are still needed if high-purity outputs are desired since the performance might worsen due to pressure drop and concentration polarization [16]. Hence, it could be an efficient approach to produce high-concentration CO_2_ via a hybrid system, which combines m-DAC and other CO_2_ capture systems, although more research is needed in this area.

With m-DAC, the contaminants impacting CO_2_ purity are gases like O_2_ and N_2_, not NO_x_ and SO_x_, which are picked up by flue gas capture. It seems only agricultural applications can use dilute CO_2_ [5]. Biological fixation through photosynthetic organisms is the planet’s natural method for CO_2_ recycling; photosynthetic microorganisms offer fast reproduction, adaptability, and efficient conversion of CO_2_ into sugars and typically require low CO_2_ (<~40%) concentrations [16,109] (Table S2). With currently commercially available membranes, the 2% CO_2_ concentration could be of interest for the intensification of greenhouses or algae ponds. Captured high-purity CO_2_ can also be used to enhance oil recovery. It can be used for welding, dry ice, soda, and feedstock for greenhouses and other agricultural installations. Furthermore, CO_2_ can be used for conversion to several commercial chemicals such as ammonia for fertilizer, plastics which reduce the use of petrochemical products, formic acid, synthetic fuels by methanation, and methanol, to name a few [109]. In that event, multistage membrane units or hybrid processes could be of interest [29]. Although most electrochemical reduction of CO_2_ uses pure CO_2_, recent studies have shown that CO_2_ reduction to CO can be achieved with low CO_2_ purity, indicating another realistic use for m-DAC CO_2_ [110]. The CO produced can be used to produce useful hydrocarbons for downstream applications. For all the aforementioned uses, the source of energy (renewable or not) for conversion and the processes’ emissions should be considered to determine the overall environmental impact and the extent of negative emissions.

Currently, since m-DAC is rather expensive with current membranes, the most attractive selling point is the modularity. Fujikawa et al. showed through process simulation that a 4-stage separation module using current highly permeable membranes can fit into 0.01 m^3^ and capture 1 kg-CO_2_ per day [12]. Dittmeyer et al. proposed DAC-integrated AC units that could convert captured CO_2_ into hydrocarbon fuels. Their paper provided perspective examples of towns fueling their cars using the fuels generated by negative emission technologies. This approach could add an incentive for the public to get involved in DAC technology [25]. Small-scale m-DAC installations have the potential to be installed in office buildings and schools where the CO_2_ concentration reaches up to 1000 ppm [34]. Areas with elevated CO_2_ concentrations compared to the atmosphere would increase the capture efficiency of m-DAC. Moreover, the location independence of m-DAC allows for installation near CO_2_ storage and recycling sites. This process can avoid the construction of pipelines needed to transport the CO_2_ recovered from location-dependent capture sites to storage sites [7]. This also avoids the energy required to compress the gas for transport. Using the previously mentioned utilization of CO_2_ as an agricultural feedstock as an example, m-DAC modules could become a standard installation in every greenhouse.

## 6. Perspective

The m-DAC process has been highlighted as a promising complementary technology to sorbent-based DAC. Currently, the major barriers facing m-DAC’s full-scale implementation include membrane material performance, membrane fabrication, and system process development [12]:

(1) Membrane materials

The performance of membrane materials is significantly affected by the trade-off of permeance/selectivity as well as plasticization and physical aging. More research needs to be conducted on m-DAC materials to meet the criteria of CO_2_ permeance > 10,000 GPU and CO_2_/N_2_ selectivity > 30. Manipulation of the physical (mainly free volume) and chemical (mainly reactivity with CO_2_) membrane environment to improve performance via novel chemistries and preparative methods should continue to be an active area of research [86]. Besides employing novel chemistries to prepare polymers via improving the chain rigidity by introducing more inflexible units, the development of polymer nanocomposites through different approaches, e.g., “in-situ micelle-induced blending” and “gel mixed matrix compositing”. Additionally, using proper nanomaterials, such as selected functionalized MOF, silica, and GO, looks promising in terms of CO_2_/N_2_ separation performance, cost, synthesis, and feasibility. Notably, some inorganic/organic fillers could not only help tune the free volume of the polymer matrix but also increase CO_2_ solubility or create a coefficient path [98].

In addition, exploiting facilitated transport mechanisms can also provide effective solutions for overcoming the performance trade-off effect [57]. Low-cost and high-performance membranes for DAC are required by industry. Hence the exploration of cheaper and affordable materials should always be pursued.

(2) Membrane fabrication

To meet the performance expectation of DAC and be employed in large-scale applications, membrane materials should be capable of forming thin membranes with selective layers less than 1 µm to enable high gas permeance and be packaged into large spiral wound or hollow fiber membrane modules to maximize the surface area to volume ratio [46]. In this sense, the nanofillers for mixed matrix membranes need to be carefully chosen and appropriately dispersed. Polymers that are not solvent-processable or prepared at high temperatures should attract less attention. It seems that commercially available Pebax (PEO-based polymers) and PVAm-based facilitated transport membranes are good candidates for selective coating layers [64,79].

(3) System and process

Improvements to the process durability of m-DAC systems against the typical, high TRL (Technology Readiness Level) separation operations, such as adsorption, absorption, and cryogenic distillation, should be made. Compared to CO_2_ separation from point sources, rather than impurities such as SO_x_, NO_x_, H_2_S, light hydrocarbons, or aromatics, the main concern of feed gases for m-DAC is water [111]. As a condensable gas, it could lead to an increase in free volume, causing a significant decline in performance. In most facilitated transport membranes where amine groups are used as the carriers, the oxidation of carriers by oxidizing gases (mainly O_2_) resulted in the deterioration of membrane performance [111]. The effective pre-treatment of feed gases and exploration of anti-oxidized carriers could help to solve the problem.

Because of the demand for CO_2_ at high concentrations, more research needs to focus on multistep and hybrid separation systems [28]. Also, the testing of these systems should be conducted physically with the support of computer modeling. More investigation is needed on process cost analysis since DAC conditions are different from those operating in typical CO_2_ capture, and process cost reductions make the process feasible. Advancements in membrane technology and m-DAC system tunability should be conducted with future downstream applications in mind, which may require a focus on CO_2_/O_2_ selectivity and permeate purity [9,12,28]. At the same time, more attention should be brought to using lower purity CO_2_ in different utilization pathways with a focus on mixtures of O_2_/N_2_/CO_2_ typical of m-DAC [111].

Although most membrane materials do not currently meet the performance criteria for m-DAC, these improved membranes should be further investigated in conjunction with current DAC methods to potentially create more efficient hybrid capture systems. For example, m-DAC can be used as a first separation stage before solid sorbents do the heavy lifting of selective capture. Other innovative ideas, such as membranes used as contactors for liquid solvents or a support structure for solid sorbent monoliths in DAC adsorbers, will inspire researchers to leverage the advantages of the polymeric membrane in DAC processes.

In addition, as suggested by Castro-Muñoz et al., membrane performance should be measured in GPU for permeance rather than Barrer for permeability for standardization across experiments with variability in membrane thickness [26].

Ultimately, the focus of new research should be on increasing membrane permeance, optimizing selectivity, increasing scalability, decreasing cost, and use of hazardous materials, which are commonly associated with DAC methods currently in use. Future m-DAC systems should provide life cycle analyses to finitely determine the extent of negative emissions and operational cost to better illustrate the feasibility of using membranes for direct air capture.

## 7. Conclusions

Increasing CO_2_ levels in the atmosphere and 2050 Net Zero Emission requirements have aroused many research efforts in exploring efficient negative CO_2_ emission technologies to capture CO_2_ from the atmosphere. In the past decades, the intrinsic advantages of membrane technology have promoted its application in CO_2_ capture from point sources. Previous efforts in membrane technology have shown the possibility of membrane processes being considered as a new approach to DAC. Due to its great potential for unprecedented improvements in capture cost, installation applications, and environmental impact compared to the current sorbent-based DAC, it is believed that m-DAC will become an important addition to the portfolio of methods aimed at realizing ubiquitous CO_2_ capture and could change the outlook on CO_2_ capture in the near future. To promote the practical applications of m-DAC, challenges should be overcome through the intersection and application of chemistry, materials science, and engineering. The research on membrane materials is fundamental as the efficiency of m-DAC systems is heavily reliant on the permeance and selectivity of the membranes.

In this mini-review, the required properties of membranes for DAC, process parameters for modeling/system design, potential materials for membranes, and applications of m-DAC have been outlined. Particularly, we summarize the advances up to 2023 in high permeability polymer-based membrane materials for CO_2_ separations which could be potential DAC membrane materials, including polymeric membranes, mixed matrix membranes, and CO_2_-facilitated transport membranes. A number of representative examples of recent advances are highlighted, followed by a brief perspective on the direction of future research and development. This work sheds light on the progress of m-DAC and encourages further research at the intersection of disciplines, such as polymer chemistry, inorganic chemistry, nanoscience and technology, and chemical engineering, drawing an inspiring picture of membranes that are more robust, have lower cost and higher CO_2_ separation performance.

## Figures and Tables

**Table 1:** The m-DAC separation outcomes with different pressure ratios [12]. Data for the Pressure ratio of 20 is summated while the pressure ratio of 25 is not (each stage).

	Pressure Ratio = 20 Permeate Pressure = 5 kPa					Pressure Ratio = 25 Permeate Pressure = 4 kPa				
Number of Separation Stages	1	2	3	4	Total	1	2	3	4	Total
CO_2_ concentration in permeate (%)	0.6	2.9	10.8	29.8	-	0.7	3.9	15.5	42.4	-
Membrane area (m^2^/kg-CO_2_/day)	2.57	(0.47)	(0.12)	(0.03)	3.19	2.15	0.35	0.08	0.02	2.6
Energy required for vacuuming (kWh-CO_2_/day)	12.7	(2.4)	(0.7)	(0.2)	16.0	11.6	1.9	0.5	0.2	14.2
CO_2_ emission related to the energy production (kgCO_2_^emitted^/kgCO_2_^captured^)	0.48	(0.09)	(0.02)	(0.01)	0.6	0.44	0.07	0.02	0.01	0.54

**Table 2:** Selection of recent promising membrane materials showing CO_2_ permeabilities ≥ 1000 Barrer or with CO_2_/N_2_ Selectivity’s ≥ 30 at temperatures ≤ 35 °C. Inorganic membranes have not been included. Some additions are not mutually exclusive and could belong to several categories. Data is from pure gas permeation measurements. A more complete summary can be found in Table S1.

Polymeric Membranes				T (°C)	Pressure (kPa)	CO_2_ Permeability (Barrer)	Gas Selectivity (CO_2_/N_2_)	Ref.
Type	Polymers							
Polymer membranes	Copolymers	KAUST-PI-1		35	200	2389	33	[40]
		PIM-BTrip (160 μm)	Aged 490 days	25	100	6060	31.0	[41]
			Aged 120 days	25	100	6040	30.2	[41]
		SFX-PIM-33 (Aged 130 days)	(Aged 130 days)	25	200	1848	30.8	[42]
		BPM-50		35	350	4883	43	[43]
		VAP7		30	100	1370	32	[44]
		PTCNSi(OMe)_3_		20–22	100	2000	35.7	[45]
		Polaris™ gen1		-	-	1000 (Commercially Available)	50	[46]
		PolyActive™/85		-	-	1480	55	[47]
	Copolymers with post modification	TZ-PIM-1		25	440	~3000	~30	[48]
		AO-PIM-1 + Methanol		35	200	1153	35	[49]
		MTZ100-PIM *		25	350	1391	22.2	[50]
		Thioamide-PIM-1 + Ethanol		25	100	1120	30.3	[51]
		cPIM-1		25	200	3739 ± 32	34.9	[52]
Mixed matrix membranes		6FDA-durene/Si-5		25	200	3785	31	[53]
		PIM-MFI3		25	100	2530	30	[54]
		Pebax-2533/ZIF	35 wt%	25	200	1287	32.3	[55]
		SPEEK/MIL-101 (Cr) 40 wt% *		30	100	30	40	[56]
		SPEEK/S-MIL-101 (Cr) 40 wt% *		30	100	35	41	[56]
		UiO-66-CN@sPIM-1 *		25	140	16,121.3	27	[57]
		PDMS-SAPO-34 (PM-30 wt%)		25	2000	5753	31	[58]
		PIM-1/GO		30	400	6169	123	[59]
		CNT-ZIF-8-PDMS		25	100	8705	45.6	[60]
		PAO-PIM-1/NH2-UiO-66	7 wt%	35	100	3825	30.0	[61]
		PEO/HPNs	0.5 wt%	35	100	~1400	~41	[62]
			1 wt%	35	100	~1900	~44	[62]
Facilitated transport membranes		Pebax [C4MIM][Gly] 20 wt%		25	100	~1100	~110	[63]
		C(30)-P(1:1)		25	200	~1650	~55	[64]
		Pebax-PEI-MCM-41-20		25	100	1521	102	[65]
		15 wt% ([Cu(6)]2+@13X)/6FDA-Durene		35	200	~1034	38.3	[66]
		Pebax 1657/MWNTs-NH2/GTA (P10CN1G25)		35	700	1408	~40	[67]
		Pebax 1657/SG 20 wt%		25	200	~1200	~55	[68]
		CA/PM-4 (1:3 wt%)		35	300	3000	59	[69]
		PIM-Py-Cl 15 wt%		25	200	4959.8	42	[70]
		PIM-Py-Ac 15 wt%		25	200	6204.8	62	[70]
		PIM-Py-BF4 15 wt%		25	200	5584.3	46	[70]

The membranes marked with a * do not meet the table’s criteria through pure gas testing but do meet the criteria under mixed gas conditions or humidified conditions which are covered later.

## Data Availability

Data openly available in a public repository that issues datasets with and without DOIs.

## Supplementary Materials

The following supporting information can be downloaded at: https://www.mdpi.com/article/10.3390/membranes14020030/s1, Table S1: Summary of recent promising membrane materials showing permeabilities ≥ 1000 Barrer or permeances > ~1000 GPU or greater with CO_2_/N_2_ Selectivity’s ≥ 20 at temperatures ≤ 35 °C; Table S2: CO_2_ purity requirements for downstream applications. References [112,113,114,115,116,117,118,119,120,121,122,123,124,125,126,127,128,129,130,131,132,133,134,135,136,137,138,139,140,141,142,143,144,145,146,147,148,149,150,151,152,153,154,155,156,157,158,159,160,161,162,163,164,165,166,167,168,169,170,171,172,173,174,175,176] are cited in the Supplementary Materials.
