# Supplementary material for: Membrane Separation Technology in Direct Air Capture
Source: Membranes (Basel). 2024 Jan 24;14(2):30. doi: 10.3390/membranes14020030 (PMC10889985; doi:10.3390/membranes14020030)
Supplement: Supplementary file 1 [file membranes-14-00030-s001.zip › membranes-2796374-supplementary.pdf]

Supporting information

Membrane Separation Technology in Direct Air Capture

Pavlo Ignatusha [1,2], Haiqing Lin [3,*], Noe Kapuscinsky [1,4], Ludmila Scoles [1], Weiguo Ma [1], Bussaraporn Patarachao [1] and Naiying Du [1,*]

Table S1: Summary of recent promising membrane materials showing permeabilities ≥1000 Barrer or permeances >~1000 GPU or greater with CO_2/N_2 Selectivity's ≥20 at temperatures ≤ 35 °C. Inorganic membranes have not been included. Some additions are not mutually exclusive and could belong to several categories. Data is from pure gas permeation measurements unless otherwise specified.

Polymeric Membranes			T (°C)	Pressure (kPa)	Permeance (GPU)	Permeability (Barrer)	Gas selectivity (CO_2/N_2)	Ref
Type	Polymers							
Copolymers	PIMs (polymers of intrinsic microporosity)							
	PIM-1		30	20	-	2,300	25	75
	PIM-1 + Methanol		35	200	-	5,919	24	49
	PIM-1 (Aged 2219 days)		25	100	-	5,000	22.0	112
	PIM-7		30	20	-	1,100	26	5
	PIM-HPB	As cast	100	100	-	1,640	24.3	113
		After Methanol	100	100	-	3,800	20.0	113
		aged 145 days	100	100	-	2,390	22.3	113
	PIM+CH_3-HPB	After Methanol	100	100	-	2,620	21.4	113
		aged 147 days	100	100	-	1,630	27.3	113
	PIM-Br-HPB	After Methanol	100	100	-	2,130	23.0	113
		aged 146 days	100	100	-	1,430	26.5	113
	PIM-CN-HPB	aged 147 days	100	100	-	1,300	24.7	113
	DPPD-TMPD		30	300	-	1,600	21.9	114
	TPE-PIM-50		35	200	-	1,869	21.3	115
	TPE-PIM-25		35	200	-	5,203	20.3	115

	PIM-PI-10		20-22	100	-	2,154	25.6	82
	PIM-PI-8		20-22	100	-	3,190	22.8	82
	PIM-PI-11		20-22	100	-	1,523	23.4	82
	KAUST-PI-1		35	200	-	2,389	33	40
	KAUST-PI-2		35	200	-	2,071	21	40
	KAUST-PI-7		35	200	-	4,391	20	40
	TBDA2-SBI-PI		35	100	-	1,213	24.8	116
	PIM-BTrip (160 µm)	Aged 130 days	100	100	-	13,200	25.3	41
		Aged 253 days	100	100	-	10,700	26.7	41
		Aged 365 days	100	100	-	8,020	28.6	41
		Aged 490 days	100	100	-	6,060	31.0	41
		Aged 633 days	100	100	-	4,350	34.2	41
		Aged 718 days	100	100	-	3,770	33.8	41
	PIM-BTrip (64 µm)	Aged 0 days	100	100	-	9,200	27.1	41
		Aged 120 days)	100	100	-	6,040	30.2	41
		Aged 253 days	100	100	-	5,990	31.5	41
		Aged 371 days	100	100	-	5,150	33.4	41
	PIM-HMI-Trip (135 µm)	Aged 426 days	100	100	-	16,400	20.4	41
	PIM-TFM-BTrip (176 µm)	Aged 123 days	100	100	-	22,100	20.3	41
		Aged 255 days	100	100	-	18,400	21.0	41
		Aged 367 days	100	100	-	17,000	21.5	41
		Aged 496 days	100	100	-	15,600	21.6	41
	PIM-DTFM-BTrip (112 µm)	Aged 636 days	100	100	-	14,800	20.0	41
	PIM-DM-BTrip (114 µm)	Aged 0 days	100	100	-	22,000	21.8	41
		Aged 128 days	100	100	-	12,200	23.4	41
	DFTTB	Aged 0 days	200	200	-	3,146	28.9	117
		Aged 7 days	200	200	-	2,102	27.7	117
		Aged 180 days	200	200	-	1,005	23.4	117
	ITTB	Aged 0 days	200	200	-	3,901	20.9	117
		Aged 30 days	200	200	-	2,203	22.7	117
		Aged 150 days	200	200	-	1,370	22.5	117
	PIM-Trip-TB	Aged 100 days	200	200	-	3,951	20.9	117
	PIM-SBI-Trip (Aged 501 days)		25	100	-	11,500	24.4	112
	PIM-PI-1		30	-	-	1,100	23.4	83
	PIM-PI-8i		30	-	-	3,700	23.1	83
	PIM-PI-8ii		30	-	-	2,270	22.7	83
	TPIM-1		25	200	-	1,549	28.7	118
	PAO-PIM-1		35	100	-	2,902	28	61
	NTDA-TMDAT		35	200	-	1,441	20.6	119
	BCDA-TMDAT		35	200	-	1,015	22.1	119

	TPDA-TMPD		35	200	-	2,389	22.3	119
	Bio-PITB-1-Vac	Aged 0 days	100	100	-	1,123	22	120
		Aged 100 days	100	100	-	1,008	22	120
	Bio-PITB-1-Air	Aged 0 days	100	100	-	1,352	21	120
		Aged 100 days	100	100	-	1,076	22	120
	Bio-PITB-2-Vac	Aged 0 days	100	100	-	1,201	22	120
		Aged 100 days	100	100	-	1,087	22	120
	Bio-PITB-2-Air	Aged 0 days	100	100	-	1,384	22	120
		Aged 100 days	100	100	-	1,161	23	120
	SBFDA-DMN (day 10)		35	200	-	3,049	21.3	121
	PIM-PI-12		35	200	-	3,230	24.5	121
	PIM-SBF-1	Aged 0 days	100	100	-	8,850	26	78
		Aged 2088 days	100	100	-	2,410	27.5	78
	PIM-SBF-2	Aged 1295 days	100	100	-	3,870	23.3	78
	PIM-SBF-3	Aged 0 days	100	100	-	10,900	23.5	78
		Aged 1294 days	100	100	-	4,850	22.8	78
	CTB1-DMN		35	200	-	1,661	21.8	122
	PIM-EA(H2)-TB		25	100	-	1,445[a]	29.25	123
	PIM-EA(H2)-TB		25	100	-	1391	26.20	123
	PIM-Trip-TB (Aged 470 Days)		25	100	-	3,951	21.0	124
	PIM-CO15		30	20-70	-	1,070	26.8	125
	PIM-CO15		30	20-70	-	2,000	24.1	125
	PIM1-CO15-75		30	20-70	-	2,570	23.4	125
	PIM1-CO15-50		30	20-70	-	4,600	21.9	125
	PIMCO1-CO15-50		30	20-70	-	5,400	22.5	125
	PIMCO2-CO15-50		30	20-70	-	5,300	20.4	125
	PIMCO6-CO15-50		30	20-70	-	3,800	22.4	125
	PIMCO19-CO15-50		30	20-70	-	3,400	22.7	125
	PHPIM101		30	100	-	4,627	25.1	126
	PHPIM081		30	100	-	3,237	27.5	126
	PHPIM061		30	100	-	2,718	24.1	126
	PHPIM041		30	100	-	1,850	26.1	126
	PIM-bpy-5		35	200	-	5,141 ± 310	21.2 ± 1.3	77
	PIM-bpy-10		35	200	-	4,917 ± 295	22.0 ± 1.5	77
	PIM-bpy-15		35	200	-	4,035 ± 262	22.2 ± 1.7	77
	SFX-PIM-33		25	200	-	3,595	23.5	42
	SFX-PIM-33 (Aged 130 days)		25	200	-	1,848	30.8	42
	SFX-PIM-25 (Aged 130 days)		25	200	-	2,120	20.2	42

[a] Mixed gas. v CO_2: v N_2= 15 : 85

	PEO containing polymer						
	$PDMS^5/O_2^{0.6}/Pebax^{0.3}$	25	200	3,574 ± 413	-	22.5	32
	$PDMS^5/O_2^{0.7}/Pebax^{0.3}$	25	200	3,292 ± 239	-	37.0	32
	$PDMS^5/O_2^{0.8}/Pebax^{0.3}$	25	200	2,742 ± 41	-	42.9	32
	$PDMS^5/O_2^{0.9}/Pebax^{0.3}$	25	200	1,511 ± 85	-	53.1	32
	$PDMS^5/O_2^{1.0}/Pebax^{0.3}$	25	200	1,249 ± 155	-	71.9	32
	$PDMS^5/O_2^{0.7}/Pebax^{0.2}$	25	200	3,233 ± 396	-	21.1	32
	$PDMS^5/O_2^{0.7}/Pebax^{0.3}$	25	200	3,292 ± 239	-	37.0	32
	$PDMS^5/O_2^{0.7}/Pebax^{0.4}$	25	200	2,516 ± 293	-	47.7	32
	$PDMS^5/O_2^{0.7}/Pebax^{0.5}$	25	200	1931 ± 147	-	30.0	32
	Pebax 1657/67 on Plasma Treated PDMS	25	100	1,140[b]	-	44	127
	Pebax 1657/45 on Plasma Treated PDMS	25	100	1,614[b]	-	37	127
	Pebax 1657/35 on Plasma Treated PDMS	25	100	2,022[b]	-	29	127
	HPEO2-800	35	70-200	1,000	-	39	128
	BPM-0	35	350	-	1,711	44	43
	BPM-10	35	350	-	2,036	44	43
	BPM-20	35	350	-	2,674	46	43
	BPM-30	35	350	-	3,277	44	43
	BPM-40	35	350	-	4,169	44	43
	BPM-50	35	350	-	4,883	43	43
	EO-21 (DAmPEG-0.0064)	22	500	1,310[a]	-	33	129
	EO-21 (DAmPEG-0.0016)	22	500	1,264[a]	-	37	129
	EO-21 (DAmPEG-0.0032)	22	500	1,308[a]	-	35	129
	EO-3 (TMC- 0.0094)	22	110	1,287[a]	-	39	129
	EO-3 (TMC- 0.0104)	22	110	~1,000[a]	-	84	129
	Thermally Rearranged Polymers						
	TPBO-0.25	35	300	-	1,213 ± 28	~21	87
	TPBO-Ac-0.25	35	300	-	1,433 ± 30	~20	87
	6FDA-SBF-PBO (420 °C)	35	200	-	1,160	21.1	88
	SPDA-SBF-PBO (450 °C)	35	200	-	1,280	20.8	88
	PI-S1-425	35	200	-	1,389	20.8	130
	TR-BMI-6F (450°C)	35	100	-	5,440	23	131
	tTR-450(tTR-PBO)	35	100	-	3,575	23.1	132
	PBI (450 °C)	25	-	-	1,624	26.2	133
	PBO-co-PPL 55 (450 °C)	25	100	-	1,805	21	134
	TOX-PIM-1	22	100	-	1,100	37	85
	Other type						

[b] Mixed gas. v CO_2: v N_2= 20 : 80

	VAP7		30	100	-	1,370	32	44
	PTCNSi(OMe)$_3$		20-22	100	-	2,000	35.7	45
	PTCNSi(OEt)$_3$		20-22	100	-	1,000	21.3	45
**Copolymers with post modification**	TZ-PIM-1		25	440	-	~3,000	~30	49
	DC-PIM4		25	350	-	1,536	25.9	135
	DC-PIM5		25	350	-	1,291	28.8	135
	PIM-UV (10 min)		35	350	-	4,560	20.2	136
	AO-PIM-1 +Methanol		35	200	-	1,153	35	49
	4% w/v D-cPIM-1-70%		25	250	7,700 ± 507	-	57 ± 15	71
	4% w/v B-cPIM-1-73%		25	250	2,900 ± 130	-	77 ± 25	71
	PIM-1-UV (in quartz)/Ozone	5 mins	400	400	-	6,960	20.7	84
		10 mins	400	400	-	6,721	25.6	84
		20 mins	400	400	-	3,781	27.7	84
		30 mins	400	400	-	2,394	28.4	84
		40 mins	400	400	-	1,364	32.4	84
	PIM-1-UV (in air)/Ozone	5 mins	400	400	-	6,007	20.9	84
		10 mins	400	400	-	6,374	21.6	84
		20 mins	400	400	-	4,374	22.1	84
		30 mins	400	400	-	1,555	26.9	84
		40 mins	400	400	-	1,535	25.6	84
		60 mins	400	400	-	1,147	30.0	84
	PIM-250	1.0 d	350	350	-	2,220	20.7	86
		2.0 d	350	350	-	1,968	25.9	86
	PIM-300	0.5 d	350	350	-	2,496	25.3	86
		1.0 d	350	350	-	3,083	30.7	86
		1.5 d	350	350	-	3,339	31.4	86
		2.0 d	350	350	-	4,000	41.7	86
	MTZ100-PIM		25	350	-	1,391	22.2	50
			25	350	-	1,674[c]	39.9	50
			25	350	-	1,780[d]	39.0	50
			25	350	-	2,057[b]	41.6	50
	Ester-Crosslinked COOH PIM-1 (200 °C)	2h	200	200	-	6,489	20.4	137
		12h	200	200	-	6,347	21.0	137
		24h	200	200	-	3,654	20.5	137
	sPIM-1		25	140	-	5,928.8 ± 111.7	23.5 ± 0.9	57
	SBFDA-DMN	Pristine	200	200	-	4,700	20.8	138

[c] Mixed gas. v CO_2: v N_2= 40 : 60

[d] Mixed gas. v CO_2: v N_2= 30 : 70

		500 °C	200	200	-	1,500	22.7	138
		600 °C	200	200	-	2,853	25.9	138
	Thioamide-PIM-1 + Ethanol		25	100	-	1,120	30.3	51
	cPIM-1/PPN$_2$-3%		25	200	-	11,511 ± 97	24.3	52
	cPIM-1		25	200	-	3,739 ± 32	34.9	52
**Matrix polymer**	30%Ag+@10%UiO-66-NH2-PIM MMM		25	200-1000	-	>15,000[e]	~30	94
	PIM-1/MOF-74-Ni		35	100	5,018	-	31	91
	PIM-1/MOF-74-Ni (Aged 8 weeks)		35	100	1,200	-	30	91
	PIM-1/NH$_2$-UiO-66		35	100	7,460	-	26	91
	PIM-1/NH$_2$-UiO-66 (Aged 8 weeks)		35	100	900	-	26	91
	ODPA-TMPDA	10wt % PS-MFI	35	100	-	2,275 ± 82[f]	26.1 ± 0.3	139
		20wt % PS-MFI	35	100	-	2,280 ± 80[f]	28.3 ± 2.1	139
		30wt % PS-MFI	35	100	-	2,397 ± 18[f]	28.8 ± 0.4	139
		10wt % ETS-10	35	100	-	1,056 ± 17[f]	33.8 ± 0.7	139
		20wt % ETS-10	35	100	-	1,225 ± 62[f]	36.6 ± 1.7	139
		30wt % ETS-10	35	100	-	1,234 ± 22[f]	34.2 ± 1.2	139
		10wt % SAPO-34	35	100	-	1,167 ± 51[f]	30.4 ± 1.0	139
		20wt % SAPO-34	35	100	-	2,248 ± 42[f]	33.2 ± 0.7	139
		30wt % SAPO-34	35	100	-	2,615 ± 34[f]	31.7 ± 0.1	139
	UiO-66-NH$_2$@PIM-1/MEEP80	10%	22	150	-	4,968[g]	22.5	92
		20%	22	150	-	5,870[g]	21.9	92
		30%	22	150	-	5,970[g]	22.5	92
	6FDA-durene/Si-1		25	200	-	1,783	29	53
	6FDA-durene/Si-2		25	200	-	1,967	29	53
	6FDA-durene/Si-3		25	200	-	2,439	30	53
	6FDA-durene/Si-4		25	200	-	3,293	31	53
	6FDA-durene/Si-5		25	200	-	3,785	31	53
	PIM-MFI3		25	100	-	2,530	30	54
	Pebax-2533/ZIF	25%	25	200	-	1,082	31.3	55
		30%	25	200	-	1,176	31.6	55
		35%	25	200	-	1,287	32.3	55
	PVC-g-POEM/ZIF-8 (40%)		35	10	-	1,195.4	26.1	140
	SPEEK/MIL-101 (Cr) 40%		30	100	-	1,623[h]	40	56
	SPEEK/S-MIL-101 (Cr) 40%		30	100	-	2,064[h]	53	56
	PAN/UIO66-NH$_2$		35	100	3,691	-	92.5	93

[e] Mixed gas. 1 : 9 in mol

[f] Mixed gas. 21 : 79 CO_2/N_2

[g] Mixed gas. 20 : 20 : 60 % mol CO_2/N_2/Dry Ar

[h] Humidified gas

	PAN/ZIF-8		35	100	3,596	-	80.3	93
	PAN/UIO66-NH_2		35	100	3,586[i]	-	90.1	93
	SAPO-34 (PM-30 wt %)		25	2000	-	5,753	31	58
	PTO		30	100	737 ± 38	-	38.0 ± 2.9	141
	PTO-U5		30	100	801 ± 11	-	39.2 ± 1.2	141
	PTO-U10		30	100	1,070 ± 76	-	41.0 ± 0.8	141
	PTO-U15		30	100	1,108 ± 16	-	38.7 ± 0.8	141
	PTO-U20		30	100	1,467 ± 42	-	34.5 ± 2.2	141
	PTO-U30		30	100	1,828 ±161	-	32.4 ± 1.9	141
	MXene/PEG (400)		25	100	1,543.13	-	30.90	142
			25	100	1,408.94[i]	-	28.18	142
	MXene/PEG (600)		25	100	1,626.99	-	32.18	142
	MXene/PEG (600)		25	100	1,912.14[i]	-	31.24	142
	EM400/MIL-101(Cr)-NH_2/TPP (60wt % TPP)		35	100	-	1,288.3	22.2	100
	UiO-66-CN/PIM-1		25	140	-	7,070.9 ± 46.2	26.7 ± 1.8	57
	UiO-66-CN@sPIM-1		25	140	-	16,121.3 ± 138.9	27.0 ± 0.6	57
	UiO-66-CN@sPIM-1		25	140	-	12,063.3 ± 106.2[i]	53.5 ± 4.1	57
	NUS-8-NH_2/PIM-1	1.6%	25	200	-	10,528[b]	25	143
		2.5%	25	200	-	10,819[b]	28.2	143
		5.6%	25	200	-	11,573[b]	30.7	143
		10.4%	25	200	-	14,638[b]	29.2	143
		13.0%	25	200	-	14,622[b]	24.7	143
		15.0%	25	200	-	14,039[b]	24.9	143
	PIM-1/UiO-66(Zr)	9.1 wt%	25	100	-	5,940	23.2	144
		16.6 wt%	25	100	-	7,610	20.7	144
		16.6 wt% + Methanol	25	100	-	9,980	21.6	144
		23.1 wt%	25	100	-	7,610	20.7	144
		23.1 wt% + Methanol	25	100	-	9,980	21.6	144
	PIM-1/UiO-66(Zr)$(CO_2H)_2$	9.1 wt%	25	100	-	4,600	21	144
		16.6 wt%	25	100	-	5,190	20	144
		23.1 wt%	25	100	-	5,300	20	144
		28.6 wt%	25	100	-	6,090	21	144
		28.6 wt% + Methanol	25	100	-	9,020	22	144
	PIM-1/UiO-66(Zr)-NH_2	9.1 wt%	25	100	-	4,810	22.3	144
		9.1 wt% + Methanol	25	100	-	8,740	22.0	144
		16.6 wt%	25	100	-	6,340	20.9	144
		16.6 wt% + Methanol	25	100	-	10,700	21.45	144

[i] Mixed gas. v CO_2: v N_2= 50 : 50

		23.1 wt%	25	100	-	5,070	20	144
		23.1 wt% + Methanol	25	100	-	9,570	23	144
		28.6 wt%	25	100	-	6,310	22	144
		28.6 wt% + Methanol	25	100	-	9,030	20	144
	PIM-1/GO		30	400	-	6,169	123	59
	PZ-10		30	100	1,219	-	41.4	145
	PZ-20		30	100	1,308	-	39.7	145
	PZ-30		30	100	1,642	-	36.7	145
	PZ-40		30	100	2,821	-	35.7	145
	PZ-50		30	100	4,474	-	32.0	145
	PZ-60		30	100	6,942	-	24.1	145
	Pebax/ZIF-8	30%	30	100	1,394	-	22.9	145
	PIM-1/SNW-1(5)		30	200	-	6,080	21.7	146
	PIM-1/SNW-1(10)		30	200	-	7,553	22.7	146
	PAO-PIM-1/NH_2-UiO-66	7%	35	100	-	3,825	30.0	61
		15%	35	100	-	4,832	28.9	61
		30%	35	100	-	8,425	27.5	61
	PAO-PIM-1/UiO-66	30%	35	100	-	8,126	22.5	61
	PIM-20ZIF		35	350	-	5,942	20	147
	UV-10ZIF		35	350	-	1,909.3	29.1	147
	UV-20ZIF		35	350	-	2,545.7	27.2	147
	UV-30ZIF		35	350	-	3,458.6	26.9	147
	PIM-1/OPS	20%	25	400	-	2,416 ± 126	21.0 ± 2.0	148
	PIM-1/OAPS	5%	25	400	-	3,266 ± 150	20.8 ± 1.8	148
		7.5%	25	400	-	1,203 ± 60.2	25.2 ± 2.2	148
	CNT-ZIF-8-PDMS		25	100	-	8,705	45.6	60
	PPM-5@MMM		30	100	-	3,443.9	22.8	149
	PPM-10@MMM		30	100	-	3,827.3	24.0	149
	PPM-15@MMM		30	100	-	1,470	21	149
	PPM-20@MMM		30	100	-	1,190	20	149
	Cardo-PIM-1	2.5 wt% o-MWCNTs	25	100	-	23,000 ± 1,200[j]	20.9	150
		7.5 wt% f-MWCNTs	25	100	-	29,000 ± 1,500[j]	24.2	150
	PIM1/6FDA-DAM/ZIF-8(20:77:3 wt%)		35	300	-	2,611 ± 106[k]	20.4 ± 0.8	151
	PDMS-PEO-SI(0.02)/CHNs(1.6)/PAN		20	200	2,860	-	28.2	152
	PIM-1A/NanoMIL-101 (18% wt filler)		25	100	-	2,830	21.3	153
	PIM-1A/MIL-101A	18% wt filler	25	100	-	3,570	21.6	153

[j] Mixed gas 1:1 CO_2/N_2

[k] Equimolar CO2/N2 Mixture (10/90cm^3(STP)min^{-1})

		39% wt filler + Ethanol	25	100	-	22,500	20.6	153
		18% wt filler	25	100	-	4,080	21.9	153
	PIM-1B/MIL-101B	30% wt filler	25	100	-	8,960	21.1	153
		30% wt filler+ Methanol	25	100	-	11,600	20.0	153
		47% wt filler	25	100	-	7,550	20.3	153
	PIM-1B/NH_2-MIL-101	18% wt filler	25	100	-	7,230	20.0	153
		18% wt filler + Methanol	25	100	-	10,200	21.0	153
	PPUN-28.6		25	100	1,320	-	30.8	154
	PEO/1% HPN		35	100	-	~1,900	~44	62
	PEO/0.5% HPN		35	100	-	~1,400	~41	62
**Polymer net work**	XS13-550	Aged 15 days	30	200	-	8,282.2	23.5	155
		Aged 30 days	30	200	-	6,215.6	27.9	155
		Aged 60 days	30	200	-	5,658.4	28.1	155
		Aged 240 days	30	200	-	5,286.5	28.1	155
	PEGMEA:PEGDA:PEGDME	9:1:10	35	350	-	1709	40.9	99
		8:2:10	35	350	-	2178	47.5	99
		7:3:10	35	350	-	2980	45.7	99
		5:5:10	35	350	-	2463	52.1	99
		3:7:10	35	350	-	2365	61.4	99
		0:10:10	35	350	-	1767	65.9	99
		7:3:3	35	350	-	1409	46.7	99
		7:3:5	35	350	-	2095	47.1	99
		7:3:7	35	350	-	2461	47.1	99
		7:3:10	35	350	-	2980	45.7	99
**Polymer Blends**	Pebax2533/PEG-b-PPFPA $P2_{1-1.5}$ (60wt %)		35	350	3,330	-	22	156
	Pebax2533/PEG-b-PPFPA $P2_{1-4.3}$ (60wt %)		35	350	1,970	-	22	156
	Pebax2533/PEG-b-PPFPA $P2_{1-7.6}$ (60wt %)		35	350	1,650	-	25	156
	PEBAX/PPEGMEA_70		35	100	-	1,388.3 ± 3.0	46.7 ± 0.6	98
	Ultem-PIM	10:90	35	350	-	2,876.6	20.0	157
	Ultem-PIM	5:95	35	350	-	3,275.9	21.1	157
	PIM-1/Matrimid	90:10	35	350	-	1,953 ± 4.8	20 ± 1.7	158
	PIM-1/Matrimid	95:5	35	350	-	3,355 ± 1.8	20 ± 0.3	158
	PIM-1/6FDA-DAM (10:90 wt%)		35	300	-	2,184 ± 152[k]	21.4 ± 1.4	151
	10 wt% PBP-menm		35	100	-	2,988 ± 365[b]	25.5 ± 0.8	159
	r-200 %PEBA		25	200	2,371 ±179	-	44.9 ± 1.2	160
	PPB-1.5		30	200	-	1,620.0	23.8	161
	PPB-3		30	200	-	1,577.4	27.8	161

	PPB-5		30	200	-	1,552.6	29.3	161
	PIM-1-10% MEEP100		22	160	-	~5,300[g]	~24	162
	PIM-1-25% MEEP100		22	160	-	~3,000[g]	~28	162
	PIM-1-10% MEEP80		22	160	-	~3,300[g]	~24	162
	PIM-1-25% MEEP80		22	160	-	~3,100[g]	~26	162
	cPIM-1/Torlon	90:10	35	350	-	1,013	24	163
	cPIM-1/Torlon	95:5	35	350	-	1,382	20.5	163
**Commercial polymers**	Polaris™ gen1		-	-	-	1000	50	46
	PolyActive™/85		-	-	-	1480	55	47
	Pebax-1657/PDMS-PEO/PAN		30	300	2,142	-	36	164
	PolyActive™($PA_{1.25}$)		35	50	3,555	-	40	165
**Facilitated transport polymeric membranes**	Pebax-[C_6MIM][Gly]-20		25	200	-	~1,500	~95	63
	Pebax [C_6MIM][Gly] 30% wt		25	100	-	~1,900	~60	63
	Pebax [C_4MIM][Gly] 20% wt		25	100	-	~1,100	~110	63
	C(30)-P(1:1)		25	200	-	~1,650	~55	64
	C(30)-P(2:1)		25	200	-	~1,200	~60	64
	TMC/DNMDAm/DGBAmE		22	110	1,612[a]	-	138	166
	Pebax-PEI-MCM-41-15		25	100	-	1,015	94	65
	Pebax-PEI-MCM-41-20		25	100	-	1,521	102	
	15% wt([Cu(6)]2+@13X)/6FDA-Durene		35	200	-	~1,034	38.3	66
	Pebax 1657/MWNTs-NH_2 /GTA (P10CN1G25)		35	700		1,408	~40	67
	Pebax 1657/SG 20 wt %		25	200	-	~1,200	~55	68
	44.4 wt% HMMP-1-PVAm		25	200	1,544[a]	-	252	167
	PVAm/PEI-g-ZIF-8(5:40:1)		25	300	1,990 ± 148[a]	-	79.9 ± 2.0	168
	PIL-IL/GO (410 ppm CO2 feed)		22	100	3,092[a]	-	1189	30
	DNMDAm-CD0.20/TMC		25	150	2,792[a]	-	171	169
	PIP-CMC/TMC		25	150	~1,479[a]	-	119	170
	PIP-CMC/TMC		25	150	~1,278	-	89	
	PVA/amine modified SiO_2/[bmim][Tf2N]		30	200	3,016	-	62.08	102
	CA/PM-4 (1:0.5 % w/w)		35	300	-	2,000	44.4	69
	CA/PM-4 (1:1 % w/w)		35	300	-	2,392	51	
	CA/PM-4 (1:3 % w/w)		35	300	-	3,000	59	
	10% MOF-303		35	300	-	6,602.8 ± 256.2[b]	25.6 ± 0.9	171
	PIM-Py-Cl 5%		25	200	-	3,393.3	35	70
	PIM-Py-Cl 10%		25	200	-	4,188.3	39	70

	PIM-Py-Cl 15%	25	200	-	4,959.8	42	70
	PIM-Py-Ac 5%	25	200	-	3,722.9	38	70
	PIM-Py-Ac 10%	25	200	-	4,690.8	47	70
	PIM-Py-Ac 15%	25	200	-	6,204.8	62	70
	PIM-Py-BF_4 5%	25	200	-	2,991.6	32	70
	PIM-Py-BF_4 10%	25	200	-	4,693.7	46	70
	PIM-Py-BF_4 15%	25	200	-	5,584.3	46	70

#

Table S2: CO_2 purity requirements for downstream applications.

**Post Capture Application**	**Required CO_2 Purity**	**Ref**
Geological Sequestration	>98 %	105
Oil Recovery	>99.9%	172
Carbonation (e. g. Beverage)	>99.9%	173
Welding (industrial)	>99.5%	174
Chemical reduction (research)	>99.99%	174
Algae Utilization	<40%	109,175
Agriculture feedstock (e. g. Greenhouse CO2 Supplement)	1000-1500 ppm	176
